# Supplementary material for: Emotion-body connection dispositions modify the insulae-midcingulate effective connectivity during anger processing
Source: PLoS One. 2020 Feb 13;15(2):e0228404. doi: 10.1371/journal.pone.0228404 (PMC7018059; doi:10.1371/journal.pone.0228404)
Supplement: Attachment — Submitted filename: Response_To_Reviewer.docx [file pone.0228404.s001.docx]

PONE-D-19-19373
Emotion-body connection dispositions modify the insulae-midcingulate effective connectivity during anger processing.
PLOS ONE

Dear Dr Dhamala,

first of all, we thank the reviewer for his/her useful comments.

We have answered all the questions in reviewing the manuscript.

Respectfully yours,

Viridiana Mazzola, PhD, PsyD

Reviewer #1:

We changed the title.

Line 76: We defined the MCC abbreviation.

Line 111: We clarified the approach we used.

Line 112: We did it.

Lines 113-114: We did it.

Line 114: We clarified this point.

Line 115: We rearranged the last paragraph of the Introduction according to the reviewer’s suggestion.

Lines-129-130: We Due to the small size of the groups, we preferred to better balance the number of the participants within each group in order to avoid any potential confounding.

We added the years of schooling we recorded. Since in the present study we did not collect genetic data, we did not consider the variable “ethnicity”. On the other hand, the culture (for example western vs eastern) we agree that it can have an impact on the emotional processing. All the participants have the same cultural background, i.e. Italians.

Line 175: We defined the units.

Line 180: We used both these standard fMRI sequences as we did in our previous studies employing the same fMRI task in order to keep a continuity (Mazzola et al. 2013, 2016). We thought that both the two sequences were sufficiently optimal to balance the sensitivity of fMRI to stimulus-correlated motion and spatial resolution for cortical regions (not subcortical structures) according to our regions of interest. Nevertheless, we agree to reconsider this issue for the next fMRI studies. We thank the reviewer for this point.

Lines 232-234: We did it.

Lines 240-241: We corrected this point.

Line 250: We decided to use either left or right MCC according to a degree of lateralization observed in insula-MCC connectivity and salience network (Cauda et al, 2011, 2012; Taylor et al. 2009).

We added all the necessary details about the spectral DCM.

Cauda, F., D'Agata, F., Sacco, K., Duca, S., Geminiani, G., Vercelli, A., (2011). Functional connectivity of the insula in the resting brain. Neuroimage 55, 8–23.
Cauda, F., Costa, T., Torta, D. M. E., Sacco, K., D’Agata, F., Duca, S., … Vercelli, A. (2012). Meta-analytic clustering of the insular cortex. Characterizing the meta-analytic connectivity of the insula when involved in active tasks. NeuroImage, 62(1), 343–355. <http://doi.org/10.1016/j.neuroimage.2012.04.012>

Taylor, K. S., Seminowicz, D. A., & Davis, K. D. (2009). Two systems of resting state connectivity between the insula and cingulate cortex. Human Brain Mapping, 30(9), 2731–2745. <http://doi.org/10.1002/hbm.20705>

Line 317: We clarified it.

We improved the resolution of figure 2.

We defined the abbreviations in Fig. 3.
