# Supplementary material for: Emotion-body connection dispositions modify the insulae-midcingulate effective connectivity during anger processing
Source: PLoS One. 2020 Feb 13;15(2):e0228404. doi: 10.1371/journal.pone.0228404 (PMC7018059; doi:10.1371/journal.pone.0228404)
Supplement: Attachment — Submitted filename: Response_To_Reviewer.docx [file pone.0228404.s002.docx]

PONE-D-19-19373R1
Emotion-body connection dispositions modify the insulae-midcingulate effective connectivity during anger processing.
PLOS ONE

Dear Dr Dhamala,

we thank again the reviewer for his/her useful comments.

We have answered all the questions in reviewing the manuscript.

Respectfully yours,

Viridiana Mazzola, PhD, PsyD

Reviewer #1:

*We are sorry we did not fully addressed the reviewer’s comments. We believe that thanks to the reviewer’s comments our manuscript has been improved.*

- Line 111: Please clarify which approach was used for DCM analysis – whether it was Bayesian Model Selection (BMS) or Bayesian Parameter Average (BPA) or BMS followed by BPA. I think it’s only BMS which provides optimal model as mentioned in the following sentence. Also, please define the abbreviation BPA here.

*As written in Model comparison section, inference on family model structure was performed using Fixed Effects Bayesian Model Selection family inference analysis (FFX BMS) (Penny et al., 2010), then a FFX analysis of the model parameter estimates was performed using Bayesian Parameters Averaging (BPA)(Acs and Greenlee, 2008, Garrido et al., 2007; Neumann and Lohmann, 2003).*

*We briefly added in introduction section.*

- Lines 113-114: Please define the term ‘effective connectivity’ before using this term in the Introduction.

*We did write only ‘mid-posterior insulae-MCC connectivity’.*

- Line 114: Its confusing whether authors used traditional DCM technique or spectral DCM approach, or both - one of task-based condition and the other for resting-state. If authors used both, then please clarify the rationale behind using two approaches rather than using traditional DCM for both.

*With respect to resting state fMRI, spectral DCM has been found to be more accurate and more sensitive to group differences compared to stochastic DMC (Razi et al 2015). On the other hand, for task-related fMRI the most commonly and validated methods to investigate context-related perturbations of effective connections between brain regions is a basic deterministic DCM approach irrespective of group differences (Friston et al., 2003). Accordingly, we used both the approaches.*

*We added this rational in the methods section (see line 271.)*

- Line 115: It’s not clear how “according to model” phrase is used by the authors to define expected engagement of specific connectivity because the whole idea behind defining a model or model space is to test all the hypotheses defined by each model. Authors provided useful literature in earlier parts of the ‘Introduction’, so I would suggest to set up the hypothesis and expected findings based on literature and then define the model space to test the hypothesis. I would recommend to rearrange and modify the last paragraph of ‘Introduction’ section.

*At line 115 of the current manuscript we cannot find this sentence. We did already changed according to the reviewer’s comment.*

- For : “Lines 129-130: Rationale behind excluding data of one subject from second group (due to technical difficulties in first group) is not clear. I believe that you do not need to always have equal number of participants to perform DCM”, again I am not sure about the potential confounds and selection of excluded subject from DCM analysis. For DCM analysis, there is no need to make the sample size equal.

*The rationale to equal the two samples size was to balance the design and avoid any potential criticism and difficulties of interpretation of results about unbalanced design. We did perform also other analyses besides the DCM analysis, in which case an unbalanced design would have been a potential problem and a choice hard to justify in our opinion.*

*The participant not included in the second group was the one selected according to the inclusion criteria to match participants, that is, same age and gender of the excluded one due to technical problem.*

- Lines 240-241: I am still not sure about the rationale of this study based on which spectral DCM analysis was used.

*See the answer to the previous question about Line 114.*

- Line 250: Idea behind using either left MCC or right MCC is not completely addressed. Did the authors perform any tests to figure out why one side was preferred over the other? Second, if authors already defined ROIs with 6 mm radius with peak coordinates as the center, then I am not sure why authors didn’t use the full area for DCM? Third, in earlier section, authors mentioned that spherical ROIs were used so that identical ROIs can be used for DCM analysis, so I am not sure how that idea is supported by the using MCC – either for the left hemisphere or the right.

*Line 255: we cancelled the sentence that was ambiguous. We got the MNI coordinates of the MCC, as well as of the mid-posterior insula, from the ICA analysis. The resulting independent component maps* *had the both sides. Then, we included both side of the MCC separately in each model in order to respect the criteria of the parsimony in constructing DCM models and to be more precise in testing the close integration between MCC and mid-posterior insula. Indeed previous results showed a degree of lateralization observed in insula-MCC connectivity and salience network (Cauda et al, 2011, 2012; Taylor et al. 2009).* *The DCM analysis figured out which was the preferred one.*

*We utilized the same VOIs coordinates obtained from ICA analysis both for the resting-sate DCM analysis and the task-related DCM analysis.*

- I am not sure where did the authors include details/ideas about spectral DCM?

*Please, see lines 271-75*

- It’s not clear whether the results accounted for covariates such ‘age’ and ‘gender’. One of the recently published studies showed sex differences (https://onlinelibrary.wiley.com/doi/full/10.1002/jnr.24504) in the limbic network and impact of age on different cortical networks (https://www.frontiersin.org/articles/10.3389/fnagi.2017.00412/full). Moreover accounting for the effects of ‘age’ and ‘gender’ are also recommended for spectral DCM analysis.

*We did not covariate for age and gender in order to account for group differences. Modeling for within and between-group differences in terms of age and gender effect is of interest (thanks for the references!), but beyond the hypothesis of the present study. Accordingly, the rs-fMRI analysis was conceived only to check for any significant differences between groups in the regions of interest not due to task-related activity.*

- Line 317: It’s not clear how did the authors calculate and compare posterior probability (Pp) of family of models? That could be the reason authors are getting perfect values of Pp (= 1) for some of the families. Please clarify the idea behind this approach. Did the authors compare Pp of 103 models altogether and tried to figure out the best model among 103?

*The Pp reported in the manuscript of the winning family and model was calculated by the DCM12 routine implemented in SPM12 and saved in the BMS.mat file. We grouped the 102 models in three families as described in the paragraph “Specification of model architecture***”,** *line 250.*

*The Pp reported was an approximation of .9999. We changed “Pp 1” with “Pp .99”.*
